# Specific spectral sub-images for machine learning evaluation of optical differences between carbon ion and X ray radiation effects

**DOI:** 10.1016/j.heliyon.2024.e35249

**Published:** 2024-08-02

**Authors:** Raluca D. Negoita, Mihaela A. Ilisanu, Ionela N. Irimescu, Roxana C. Popescu, Mihaela Tudor, Mona Mihailescu, Eugen N. Scarlat, Ana M. Pleava, Anca Dinischiotu, Diana Savu

**Affiliations:** a Applied Sciences Doctoral School, National University of Science and Technology Politehnica Bucharest, 313 Splaiul Independentei, Bucharest, 060042, Romania; b Doctoral School of Automatic Control and Computers, National University of Science and Technology Politehnica Bucharest, 313 Splaiul Independentei, Bucharest, 060042, Romania; c Holographic Imaging and Processing Laboratory, Physics Department, Faculty of Applied Sciences, National University of Science and Technology Politehnica Bucharest, 313 Splaiul Independentei, Bucharest, 060042, Romania; d Tehnoplus Medical SRL, 1 Odobesti str, Bucharest, Romania; e Department of Life and Environmental Physics, Horia Hulubei National Institute for R&D in Physics and Nuclear Engineering, Reactorului 30, P.O. Box MG-6, 077125 Magurele, Romania; f Department of Bioengineering and Biotechnology, Faculty of Medical Engineering, National University of Science and Technology Politehnica Bucharest, G. Polizu Street, 1-7, 011061 Bucharest, Romania; g Faculty of Biology, University of Bucharest, 91-95 Splaiul Independentei, 050095 Bucharest, Romania; h Research Centre in Fundamental Sciences Applied in Engineering, National University of Science and Technology Politehnica Bucharest, 313 Splaiul Independentei, Bucharest, 060042, Romania; i CAMPUS Research Centre, National University of Science and Technology Politehnica Bucharest, 313 Splaiul Independentei, Bucharest, 060042, Romania

**Keywords:** Hyperspectral imaging, SW1353 chondrosarcoma cells, Ionizing radiations, Texture and roughness features, Machine learning, Support vector machine, Linear support vector classifier

## Abstract

Advances in radiotherapy, particularly the exploration of alternative radiation types such as carbon ions have updated our understanding of its effects and applicability on chondrosarcoma cells. Here we compare the optical effects produced by carbon ions (CI) and X-rays (XR) radiations on chondrosarcoma cells nuclei and set an automated method for evaluating the radiation-induced alterations without the need of chemical marking. Hyperspectral images (HSI) of SW1353 chondrosarcoma line carry detectable optical changes of the cells irradiated either with CI or XR compared to non-irradiated ones (REF). The differences between the spectral profiles of CI, XR and REF nuclei classes led to partitioning the HSIs into spectral sub-images. The changes are detected by support vector machine (SVM) classifiers whose performances are evaluated by the most used point metrics: sensitivity (*SEN*), accuracy (*ACC*), and precision (*PREC*), applied on spatial feature values. Specific interaction mechanisms by radiation type reveal distinct subintervals where HSIs changes are more prominent, and the classifiers perform at best. For CI the best classifiers are obtained for sub-images in the interval (424–436 nm), while for XR the best classifiers are obtained for sub-images in the interval (436–445 nm). The classifiers work better with texture features than roughness features in both cases. The classifier with the best *SEN* point metric in the testing phase is the most suitable to measure the irradiation efficiency irrespective of the radiation type. The altered nuclei are easier to discriminate when irradiated with CI than with XR. The study proves that SVM with optical data offers a rapid, automated, and label-free method for evaluating radiation-induced alterations in chondrosarcoma nuclei, thereby enabling effective analysis of extensive data.

## Highlights

• Optical effects of carbon ions CI and X-rays XR radiation on SW1353 cells nuclei, are compared in hyperspectral images HSI.
• Specific spectral profiles of CI, XR irradiated, and non-irradiated nuclei have been identified on unstained cells.
• Sub-images of HSI are suitable for machine learning ML classifiers to discriminate CI and XR from non-irradiated nuclei.
• Best classifiers are obtained for distinct sub-images by radiation type, confirming different effects.
• HSIs offer an information rich, automated, chemical-free way to evaluate the efficiency of radiation-induced alterations.

## 1 Introduction

Radiotherapy is a technique that has established itself as a “sine qua non” treatment for invasive tumors, either standing alone or in combination with surgery and chemotherapy. Radiotherapy typically uses ionizing radiation, to slow down tumor growth by altering DNA structure or to completely destroy them [1,2]. Traditionally, X rays (XR) were largely used, despite the disadvantages that they provoke inherent damages of the tissues adjacent to the tumor site and are less effective in some types of radioresistant tumors [3]. Recently, there is increased interest in heavy-particle radiotherapy, especially to use carbon ions (CI) due to its higher precision in targeting the volume of delivered energy and, consequently, with little damaging effects on the neighboring cells [4]. Moreover, due to a higher linear energy transfer, CI exhibits a higher damaging rate of DNA which overwhelms the cellular repair capacity [5,6]. Given that different types of ionizing radiation have different effects on living organisms [7], the understanding of the specific alteration dynamics is of considerable importance in cell biology and in biomedical disciplines for developing new therapies.

Over time, several in-vitro methods have been used to predict radiotherapy effects in resistant tumor management. Such as, the evaluation of DNA lesions dynamics at cellular level was done using micronucleus test [[8], [9], [10]], gamma H2AX foci quantification [[11], [12], [13], [14]], comet assay [[15], [16], [17]], etc. These methods require biochemical markers for labelling large numbers of samples, as well as human observations and interpretations of statistical data, all being time and resources consumers. A significant advance in realistically tracking the irradiation effects would be an automated system for optical images acquisition, segmentation, identification and classification of cellular and subcellular components, as well as statistical processing to detect specific changes provoked by CI or XR radiations. For these reasons, we propose a SVM assisted investigation of spectral changes of irradiated SW1353 chondrosarcoma cells from the spatial features of images recorded by the hyperspectral module - CytoViva^R^ enhanced darkfield microscope (eDFM).

Dark field microscopy is a versatile and easy-to-use tool for label-free, non-invasive observation of cultured cells in specific situations [18,19]. When used in combination with hyperspectral imaging module, it provides hyperspectral images (HSI) with high signal to noise ratio [20] including rich chemical and structural information [21,22]. A hyperspectral image provides a 3D data-cube, which contains spatial and spectral information *I* (*x, y,* λ), where *I* is intensity. Each pixel of the image provides its own spectral signature over the visible and near-infrared regions according to the local molecular composition. In the present study, HSI monitors the pattern changes of the recorded spectra giving a direct answer on nuclei alteration and the fate of tumor cell without long-time antibody staining or additional preparations. Spectral information at pixel-level is currently used to track the presence of nanoparticles inside cultured cells [[23], [24], [25]], to study physicochemical changes in biomaterials by tracking their unique spectral signatures [26], or to evaluate environmental nanotoxicology [27]. Ionizing radiation produces structural effects [28,29] and complex biochemical changes [2] which are discernible through distinctive aspects within the scattered light spectrum. Other microscopy techniques only allow an analysis of the spatial distribution of DNA damage produced by ionizing radiation [30,31].

Different types of artificial intelligence tools have been used on hyperspectral images with the aim to differentiate cancerous from non-cancerous tissues in advanced optical diagnosis of tumors [32,33], to classify gram-positive bacteria species [34], or to determine quality of food products [35].

Here we use sub-images extracted from HSI as inputs for SVM analysis to distinguish the irradiated from the non-irradiated nuclei. We preferred SVM approach because it exhibits greater performance on binary classification [36,37] compared to other popular classifiers. Because SVMs have a lower generalization error compared to other classifiers, it appears to be advantageous especially where only few training samples are available [38]. Researchers have addressed SVM models that perform better on HSI classification applied in geosciences [[39], [40], [41]], hematology [42], tissue identification [43]. SVM classifiers have promising results in solving binary comparisons problems related to various medical diagnostics [[44], [45], [46]].

Since the two types of ionizing radiations (CI and XR) have specific mechanism to interact with living organisms, the main contributions of this study are.

1 /using HSI as a tool for detecting optical changes to distinguish the irradiated nuclei from REF,
2 /pointing out optical differences between CI and XR effects as revealed by SVM evaluation,
3 /suggesting an expedited and chemical-free way to compute irradiation efficiency.

To accomplish these tasks, we selected from the HSIs the spectral subintervals that carry the optical changes specific to each type of radiation, extracted the corresponding sub-images, and assessed the spatial features which provide the best discrimination between irradiated and non-irradiated nuclei. Texture and roughness features taken on spectral sub-images of the nuclei are inputs for SVM models that perform two binary classifications: CI-irradiated versus REF, and XR-irradiated versus REF. The point metrics sensitivity (*SEN*), accuracy (*ACC*), precision (*PREC*), and *F1* score were used to evaluate the classification results, particularly *SEN* is suitable to evaluate the irradiation efficiency.

As far as we know this is the first study when SVM algorithms are applied either on HSIs, or on sub-images extracted from HSIs to distinguish irradiated from non-irradiated cells, combining spectral-spatial evaluation.

In this way, since many activities were automated, such as determining class-averaged spectra, calculating specific wavelengths, partitioning HSI into sub-images, computing spatial features, and running LSVC routines, the analysis requires minimal human operator interventions and could be integrated into a method applicable to other cell lines and types of radiation.

Because the current trend in radiotherapy is to replace XR with ion radiations such as CI, here we did a comparative study on their optical effects. Also, the SW1353 chondrosarcoma cell line was chosen because of its resilience to XR radiotherapy; generally, this line is chosen with priority for studying the effects of heavy ion irradiation.

We have shown that the spectral interval on which the analysis was performed does matter, and for this reason we have proposed a couple of rules to select the best classifiers. The number of best LSVC classifiers represents a semi-quantitative indicator of the strength of the changes produced by each type of irradiation: the more selected classifiers, the more intense the alterations, and therefore the easier their detection. We compared two types of spatial features, highlighting the importance of choosing appropriate features for accurate analysis. *SEN* point metric used so far only to evaluate the performances of classifiers, has been given here a physical interpretation, to measure the efficiency of the irradiation-induced alteration.

We can state that our analysis meets the need for a procedure capable of automatically handling large optical datasets and providing quantitative information for irradiation efficiency without the use of staining markers. In addition, due to its capacity of rapidly extracting the information embedded in the tumor cells, this method could also be promising in monitoring their behavior during several strategies in radiotherapy.

## 2 Hyperspectral imaging

### 2.1 Samples preparation

SW1353 chondrosarcoma cells (CLS Cell Lines Service GmbH, Eppelheim, Germany, Product number: 300440) were cultured in Dulbecco's Modified Eagle Medium (DMEM, Sigma-Aldrich, St. Louis MO, USA) supplemented with 10 % fetal bovine serum (FBS, Sigma-Aldrich, St. Louis MO, USA) and 1 % Penicillin-Streptomycin (Sigma-Aldrich, St. Louis MO, USA), in standard conditions of temperature and humidity (37 °C, 5 % CO_2_, 90 % humidity).

Cells at a concentration of 500000 cells/flask were seeded in T25 flasks 24 h before irradiation using different sources: 150 kV X-Rays (XSTRAHL X-Ray generator Ratingen, Germany) and respectively 95 MeV carbon ions (IRABAT Ganil Caen, Franta) at 4Gy. The 73 keV/μm low energy transfer of carbon ions were obtained using a methyl polymethacrylate (PMMA) filter placed between the beam exit area and the irradiation sample.

After irradiation, cells were detached and seeded onto 10 mm cover slip glasses at a concentration of 10000 cells/slide. Cells were incubated for 24 h to allow attachment and then fixed using 3.7 % Paraformaldehyde solution. These coverslip glasses were mounted on microscope slides using glycerol. Control cells were represented by non-irradiated SW1353 cells, prepared in the same manner, but excluding the irradiation step.

At the end of this procedure, one has samples with CI-irradiated cells, with XR-irradiated cells and non-irradiated cells (REF). Each experiment was triplicated, resulting in 3 cover slips for REF, 3 for XR, and 3 for CI, used in this study.

### 2.2 Images acquisition

CytoViva-eDFM system with hyperspectral module was used to record the images of the samples. The elastically scattered light from the samples enters the objective forming the image, while incident light is blocked leading to dark background with bright appearing details, generating a higher signal-to-noise ratio. In CytoViva-eDFM system, the white light source (Dolan-Jenner Industries, DC-950 Regulated Illuminator) is connected to fiber optic (liquid core) and then directed to the cardioid annular oil immersion dark-field condenser to reflect the aberration-free light at an oblique angle. A 60 × objective (oil immersion, 1.25 numerical aperture) collects the scattered radiation by the sample resulting in a background-free image effect, on which the micro or even nanometric details of the sample appear bright [47].

To obtain spectral information with narrow bands at each pixel of an image, the CytoViva-eDFM system is equipped with hyperspectral sensors (Pixelfly 1392 × 1040-pixel resolution, 6.45 × 6.45 μm pixel size, 7.3 to 13.5fps, 5μs-60s exposure time range, 62 % quantum efficiency), having in front a spectrophotometer (ImSpectrum V10E, Specim Finland) with a transmission diffraction grating. They acquire elastic scattered light from visible near-infrared range (VNIR; 400–1000 nm wavelength range). During image acquisition, the samples are scanned in the XY plane with a motorized stage (NanoScanZ, Prior Scientific Instruments Ltd, UK, 10 nm step size, 114 × 75 mm travel range).

Based on this configuration, high spatial resolution (107.5nm/pixel) and approx. 1.28 nm spectral resolution (468 channels between 400 nm and 1000 nm) are obtained. Parameters used to record our images were sets in Environment for Visualizing Images (ENVI) software: the 60 × objective with 0.6 numerical aperture, exposure times it is slightly adapted for each image condition, with values ranging from 0.1s to 0.3s, to avoid CCD saturation but to ensure sufficient spectral data. The field of view (maximum 696 × 696 pixels) allows the recording of large colonies with attached cells from which the nuclei were then selected under ENVI, for rapid visualization of their spectral profiles and differences by class, if any. Forty-five images were taken from different areas of all cover slips. Lamp correction was performed for all images according to the standard procedure mentioned in CytoViva User Manual, Sec.6.3. [47]. This procedure removes the effect of the lamp spectrum so that sample features are seen more clearly.

## 3 Images analysis

**Fig. 1:**
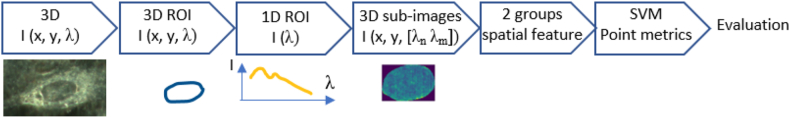
Analysis flowchart main steps (ROI – region of interest, SVM – support vector machine).

We assessed the optical differences between the nuclei of irradiated SW1353 chondrosarcoma cells compared to non-irradiated ones, separately for CI and XR on representative spectral intervals considering two binary comparisons: 1/class of CI irradiated cells vs REF class, and 2/class of XR irradiated cells vs REF class. The main steps are illustrated in Fig. 1, highlighting the passage from 3D to 1D, and back to 3D information.

**Fig. 2:**
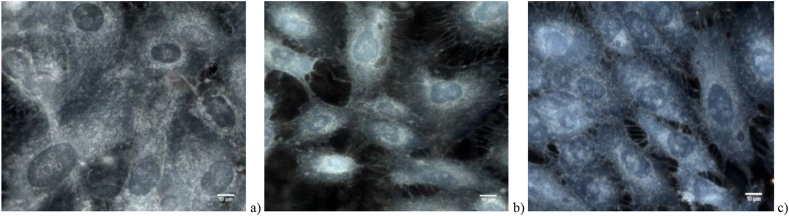
Experimental hyperspectral images with SW1353 chondrosarcoma cells a) irradiated with 4Gy CI, b) irradiated with 4Gy XR, and c) non-irradiated REF.

**Fig. 3:**
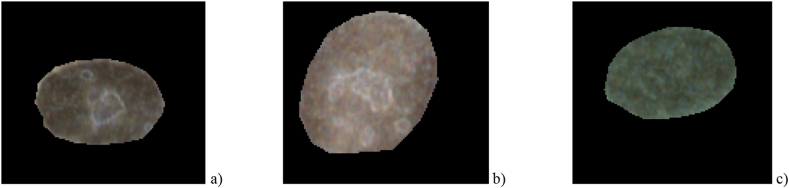
Segmented nuclei from experimental hyperspectral images: a) irradiated with 4Gy CI, b) irradiated with 4Gy XR, and c) non-irradiated.

**Fig. 4:**
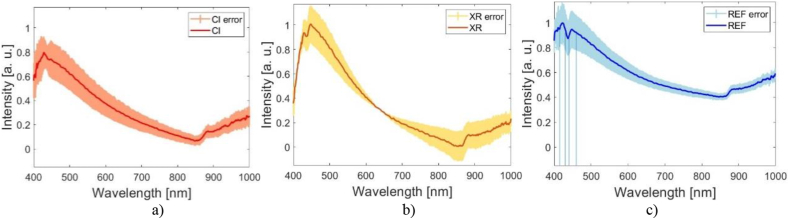
The spectral fingerprints of a) CI, b) XR, and c) REF classes with marked inflection points at 415 nm, 430 nm, 440 nm, and 460 nm.

The analysis starts with hyperspectral images (Fig. 2) that contain spatial and spectral information as 3D data cube *I* (x, y, λ), where *I* is intensity. For a compact study, the segmentation (Fig. 3) of each nucleus as region of interest (ROI) was performed using home-made scripts, which will further allow to automate all the activities that follow.

i for each nucleus, finding the nucleus spectral fingerprint as the mean values of the intensities over all pixels within the nucleus;
ii for each class, calculating the class spectral fingerprint as mean values of the intensities over all nuclei belonging to the class (Fig. 4);
iii for REF class spectral profile, finding and pointing out the characteristic wavelengths of local extrema and inflection points, and establishing the spectral subintervals delimited by the characteristic wavelengths;
iv splitting the HSI of each nucleus in sub-images corresponding to the fore-mentioned spectral subintervals;
v computing spatial features: texture and roughness parameters of the nuclei sub-images;
vi training and testing SVM models using the spatial parameters of the nuclei sub-images;
vii point metrics computation;
viii classifiers evaluation.

### 3.1 Nuclei segmentation and spectral fingerprints

In Fig. 2 are experimental images of cultured cells belonging to the investigated sample classes: a) irradiated with 4Gy CI, b) irradiated with 4Gy XR, and c) non-irradiated REF. All images highlight the advantage provided by eDFM to optically exclude the unscattered incident beam such that the nuclei areas could be identified as regions of darker color.

In-house scripts were written for nuclei segmentation and processing from images like those shown in Fig. 2. To elaborate them, we used the image processing libraries from Python 3 (OpenCV [48], Pillow [49], spectral [50], scikit-image [51], scipy [52]). First, the RGB images were imported, and the maximum number of nuclei were cut by manual segmentation of each image, creating a black and white mask for each of them. Segmentation is simplified due to the well-defined shapes and clean edges of the nuclei HSIs provided by eDFM technique, concluding that HSI under darkfield configuration can be used to identify the nucleus of the cell without any chromatographic markers, confirming other findings from the literature [53]. By multiplying the binary mask with the hyperspectral image data imported from ENVI, the nuclei areas (see Fig. 3) are identified as region of interest 3D data-cube. After segmentation, data consisted of 72 irradiated nuclei (39 CI, 33 XR) and 86 non-irradiated nuclei.

Next, spectral information of cells nuclei was extracted from segmented areas of the order of thousands of pixels. In-house developed scripts allow us to automatically obtain the spectral fingerprint of each nucleus as well as the mean spectral profile across each class (Fig. 4). Error bars are indicated in each spectral band, pointing out that although the cells response to irradiation is heterogeneous, the mean spectral fingerprints of the classes are resolved.

We designed home-made scripts based on mathematical considerations to automatically identify and mark the characteristic wavelengths for the inflection points at 415 nm, 430 nm, 440 nm, and 460 nm, local extrema at 424 nm, 445 nm (maxima), and 436 nm (minimum). The specific wavelengths which split HSI into sub-images were determined starting from the spectral profile of the REF class. Therefore, SVM has been run on sub-images taken as follows.

i over the entire range 400–1000 nm,
ii at the pointwise wavelengths corresponding to the local extrema, i.e., at 424 nm, 436 nm, and 445 nm, and
iii across the spectral subintervals between the inflection points, i.e., 415–430 nm, 430–440 nm, 440–460 nm, and between the local extrema, i.e., 424–436 nm, 436–445 nm, and 424–445 nm.

### 3.2 Spatial features

The interaction between ionizing radiation and living organisms involves a manifold of events such as oxidative damage of DNA, lipids, proteins, and many metabolites, the main effect being DNA alteration [2,54]. The hyperspectral images carry local changes of the scattered light intensity at wavelengths depending on the types of damage and irradiation. The area of modified pixels is directly linked with the number and complexity of DNA lesions from nuclei, which is proportional to the effectiveness of the radiation exposure [[55], [56], [57], [58]]. Clustered double strand breaks are formed at scale of 1–2 μm^3^ approximately [59], predominantly after the exposure to CI radiation. This affects the texture and roughness parameters of the sub-images at different spatial scales in such a way that SVM can detect.

Besides, the texture and roughness parameters are also effective on small blocks of pixels, such as segmented nuclei [60,61] to describe the local features [62]. They encode properties at different scales [[63], [64], [65]]. In-house routines (under the image processing libraries in Python 3) were written to compute texture and roughness features of the sub-images obtained from the decomposition of the original HSI (implementation using the methods from scikit-image. feature module [66] and the basic methods for array computations from NumPy [67] library of Python). Each pixel (*x,y*) of the sub-image carries the mean value of the intensity over the specified spectral subinterval; for pointwise wavelengths, it is the intensity at that wavelength.

Six texture parameters (contrast, correlation, energy, homogeneity, entropy, and dissimilarity) and seven roughness parameters (mean, root mean square, distribution skewness, distribution kurtosis, minimum, maximum, average of the five highest local maximums plus the average of the five lowest local minimum) constitute input data for SVM models that differentiate between irradiated and non-irradiated cell nuclei, for each type of radiation. For texture parameters we implemented their relationships with one distance (*dist* = 1) along directions 0°, 45°, 180°, and 315°; for roughness parameters, the predefined functions were used [[68], [69], [70]].

### 3.3 Nuclei classification

Nuclei classification aims to indicate which spectral subintervals and group of spatial features provide better discrimination by type of radiation. All computed values of texture and roughness parameters were further considered as input data for the SVM-based procedure to automatically sort into altered and not altered cells nuclei for both CI and XR cases. In each case the weight of the training data was 70 % of the total number of samples, while the remaining 30 % were for the tests.

The Linear Support Vector Classification (LSVC) [71] models with regularization parameter of 10 [72] achieved the best performances in all cases mentioned at the end of sec. 3.1; for implementation we followed the basic pipeline described at [73], but using the MinMaxScaler method from sklearn. preprocessing module to rescale the data and the StratifiedShuffleSplit method from sklearn. model_selection module to shuffle the data set before training and testing the LSVC models. The implicit parameters of each model were the default ones from the above-mentioned modules.

LSVC models were trained and tested, and their performances were evaluated using *SEN*, *ACC*, and *PREC* point metrics. They rely on true positive (*TP*), true negative (*TN*), false positive (*FP*), and false negative (*FN*) quantities (see Table 1). These point metrics are appropriate measures for evaluating the model's ability to discriminate the sub-images of irradiated nuclei from those of non-irradiated ones, that is, to detect signs of radiation-induced alterations.

**Table 1:** Confusion matrix.

		Predicted	
		Altered	Not altered
**Actual**	Irradiated	*TP*	*FN*
	Non-irradiated	*FP*	*TN*

*SEN* point metric is of particular interest when minimizing false negatives takes priority [74]. In our case, *SEN* is a measure of how well a LSVC model can correctly identify the altered nuclei due to irradiation, whatever CI or XR:

(1) $$SEN=\frac{TP}{\;TP+FN}\text{,}$$

The efficiency *E* of radiation-induced nuclei alteration by type of radiation, can be defined as:

(2) $$E=\frac{\#\;\text{altered}\;\text{nuclei}}{\#\;\text{irradiated}\;\text{nuclei}}\text{,}$$

when computing the efficiency in real cases, the images of the cells are taken only from irradiated slides, therefore there cannot exist true negatives nor false positive; it follows that the efficiency and sensitivity relationships are the same:

(3) $$E=\frac{TP}{\;TP+FN}\Rightarrow \;E=SEN$$

The straightforward conclusion is the classifier with the best *SEN* in the testing phase should measure at best the irradiation efficiency.

ACC is a measure of the overall correctness of the classification performed on quite balanced distributions like in the present work. Moreover, it is suitable for unbiased decision-making regarding both positive and negative classes [75].

(4) $$ACC=\frac{TP+TN}{TP+TN+FP+FN}$$

*PREC*, or positive prediction value, is the ratio of correct predictions for altered to the total number of nuclei that have been decided as altered:

(5) $$PREC=\frac{TP}{TP+FP}$$

Precision is a suitable metric when minimizing false positives takes priority [76].

We selected these point metrics due to distinct attributes in context of our study: *SEN* is directly related to a biophysical aspect, i.e., alteration efficiency, *ACC* gives information about the overall correctness of the evaluations; finally, higher *PREC* indicates smaller *FP*s (in the case of samples taken from non-irradiated slides the classifier must predict as rarely as possible the altered cases, ideally zero *FP* = 0).

## 4 Results and discussions

LSVC models were run for two binary comparisons: nuclei irradiated with CI (39 nuclei) versus REF (86 nuclei), and irradiated with XR (33 nuclei) versus the same REF. The distribution was therefore 125 nuclei for CI vs. REF classification, out of which 87 for training and 38 for testing, and 119 nuclei for XR vs. REF classification, out of 83 for training and 36 for testing. For each type of radiation, we study for which spectral subintervals and for which group of spatial features are obtained the best models.

The best models are understood in the sense of evaluation by the help of the point metrics mentioned by Eqs. (1), (4), (5). The models are trained and tested for each “Texture” or “Roughness” spatial feature group computed on each sub-image associated with the corresponding spectral subinterval.

Considering almost balanced test sets, the evaluation of the classification model quality relies on the following cumulative rules.

i an enabling rule, according to which the point metrics should exceed some threshold values *SEN*≥95 %, *ACC*≥91 %, and *PREC*≥91 %;
ii a priority rule, according to which firstly the greatest *SEN* is considered (meaning as low as possible *FN*s such that to correctly measure the irradiation efficiency), followed by the greatest *ACC* (we equally do not want *FN*s and *FP*s), and finally the greatest *PREC*

### 4.1 Classification considering the whole spectrum

In Table 2 are given the point metrics computed on the whole spectrum 400–1000 nm, using spatial features as two distinct groups.

**Table 2:** LSVC point metrics over the whole spectrum, by irradiation type and spatial features.

400–1000 nm	Texture			Roughness		
	SEN	ACC	PREC	SEN	ACC	PREC
**CI**	**1**	**1**	**1**	0.96	0.82	0.81
**XR**	**1**	**0.95**	**0.93**	0.96	0.76	0.76

According to the criteria, the table endorses the following remarks: i/the enabling rule is fulfilled only for texture features (bold values in Table 2), ii/all point metrics values are greater or equal for CI than for XR whatever the texture or roughness feature group, and ii/the models compute just as good the efficiency of radiation-induced nuclei alteration (no difference between CI and XR is observed).

To conclude, the best classifier for CI seems to perform slightly better than the best classifier for XR provided that they rely on the texture parameters extracted from the full HIS, therefore, to find specific differences it was necessary to partition the HSI into spectral subintervals.

### 4.2 Classification considering pointwise wavelengths

In Table 3 are the point metrics computed at specific pointwise wavelengths, using spatial features as two distinct groups.

**Table 3:** LSVC point metrics at specific pointwise wavelengths by spatial features and irradiation type.

		Texture			Roughness		
		SEN	ACC	PREC	SEN	ACC	PREC
**CI**	424 nm	0.93	0.69	0.71	0.81	0.67	0.73
	436 nm	**1**	0.69	0.69	**0.95**	0.80	0.81
	445 nm	**0.96**	0.67	0.68	**0.96**	0.77	0.76
**XR**	424 nm	0.89	0.70	0.75	**1**	0.81	0.79
	436 nm	0.81	0.70	0.79	**1**	0.84	0.82
	445 nm	0.89	0.76	0.80	0.93	0.70	0.74

Pointwise wavelengths do not meet the quality rules. Since “pointwise” means 1.28 nm bandwidth, the intervals are too short to encompass enough information for proper classification. However, if focusing on *SEN* values only, they are close to unity for CI&Texture, CI&Roughness and XR&Roughness (bold values in Table 3). The lower values of *ACC* and *PREC* indicate relatively significant *FP*s in this case.

### 4.3 Classification considering spectral sub-images

The point metrics in Table 4 are computed using the sub-images across the spectral subintervals delimited by the local extrema and inflection points as stated in the last paragraph of Sec.2.2, for each group of spatial features.

**Table 4:** LSVC point metrics over spectral subintervals by irradiation type and spatial features.

		Texture			Roughness		
		SEN	ACC	PREC	SEN	ACC	PREC
**CI**	415–430 nm	0.88	0.87	0.92	0.96	0.76	0.76
	430–440 nm	**0.96**	**0.92**	**0.93**	1.00	0.82	0.80
	440–460 nm	**0.96**	**0.92**	**0.93**	0.96	0.82	0.81
	424–436 nm	**1.00**	**0.97**	**0.96**	0.92	0.76	0.77
	436–445 nm	0.92	0.89	0.92	0.96	0.80	0.78
	424–445 nm	**0.96**	**0.95**	**0.96**	1.00	0.82	0.79
**XR**	415–430 nm	1.00	0.92	0.89	0.92	0.72	0.75
	430–440 nm	0.96	0.89	0.89	0.96	0.75	0.76
	440–460 nm	1.00	0.92	0.89	0.89	0.67	0.72
	424–436 nm	1.00	0.89	0.87	0.92	0.72	0.75
	436–445 nm	**1.00**	**0.94**	**0.93**	0.84	0.72	0.79
	424–445 nm	0.96	0.89	0.89	0.93	0.76	0.78

Among the chosen point metrics, *SEN* has the highest values across almost all spectral subintervals (Table 4); *SEN*≥0.96 in nine cases for CI and seven cases for XR, suggesting that CI radiation is more effective.

The cumulative rules are fulfilled only for texture parameters: there are four subintervals for CI, and one subinterval for XR (bold values in Table 4). These confirm the biochemical findings according to which cells are more sensitive to CI compared to XR exposure [77] and CI triggers more DNA damages and death than XR [78]. The same idea could be drawn from Table .2: the point metrics have higher (or equal) values for CI compared to XR for both groups of spatial features, when the whole spectrum is considered.

Except for the pointwise wavelengths sub-images (Table 3), where the sharpness of spectral channel (1.28 nm) does not provide enough information for reliable classifications, *PREC* values are higher for CI than for XR: *PREC*≥0.91 in six cases for CI&Texture and in only one case for XR&Texture (Table 4). Given that the REF class is the same for both binary comparisons, this indicates that *FP*s are higher for XR than for CI, that is, the altered nuclei seem to be easier to discriminate in CI case.

According to the priority rule, the best classifiers are obtained for texture group on subintervals depending on irradiation type: 424–436 nm and 424–445 nm for CI, and 436–445 nm for XR respectively. Since both best classifiers for CI and XR exhibit unity *SEN* values, the difference is marked by *ACC* metric (0.97 vs, 0.94) indicating slightly better performance for IC model, a sign that IC irradiated nuclei are more discernible than XR.

If one considers important to have low values for both *FP* and *FN*, then *F*1 score is adequate to provide a balance between *SEN* and *PREC*

(6) $$F1=\frac{2*SEN*PREC}{SEN+PREC}$$

The highest *F*1 scores (*F*1≥0.96) confirm the subintervals that better differentiate the alteration with CI compared to the alteration with XR (bold cells in Table 5). These align with what is already known that their radiobiological effects are quite distinct [4] and the amount of DNA lesions persists significantly longer in CI irradiated cells compared to XR [[79], [80], [81]].

**Table 5:** *F*1 scores over spectral subintervals by irradiation type, texture features.

		*F*1 score
**CI**	415–430 nm	0.90
	430–440 nm	0.94
	440–460 nm	0.94
	424–436 nm	**0.98**
	436–445 nm	0.92
	424–445 nm	**0.96**
**XR**	415–430 nm	0.94
	430–440 nm	0.92
	440–460 nm	0.94
	424–436 nm	0.93
	436–445 nm	**0.96**
	424–445 nm	0.92

Among all models presented in Sec.4, those that account for the entire interval classify at best both CI and XR. They give information about the efficiency of the alteration induced by irradiation, but they do not tell us anything about the differences between the two types of irradiations, that is why the partitioning into spectral sub-images is needed. Since the sub-images of HSIs are formed with integral intensity, proportional to the length of the spectral interval, the intensity differences originating from short spectral bands could be masked by summation over long intervals. Our study on specific sub-images reveals that shorter intervals exhibit significant differences between nuclei irradiated with CI and XR, although the entire interval does not.

The used radiations differ not only from the point of view of particle type, but they generate different effects when they interact with cells [4]. CI generates a greater number and more complex critical chromosomal aberrations, compared with XR [82]. CI deposits high energy density generating multiple types of DNA damages: DNA single and double-strand breaks, base damage within 1–2 helical turns, clustered double-strand breaks [59]. These phenomena change chemical composition, reflected in the nuclei spectral profiles. The differences between the effects induced by the two types of radiation can be seen in Fig. 4, the average spectral fingerprint on the CI class differing significantly from the control (lack of the second maximum). For XR class, the spectral profile contains both maxima but with their intensity values reversed.

## 5 Conclusions

The study reports the first use of hyperspectral microscopy for automated analysis of optical changes exhibited by SW1353 chondrosarcoma cells nuclei subjected to ionizing radiation. The differences among the spectral fingerprints of CI, XR, and REF classes are significant enough to provide criteria to divide HSIs into spectral sub-images.

The presence of optical changes is proved by LSVC classifiers. According to a couple of selection rules, there are more classifiers which fulfill the threshold values in the case of CI than in XR case, indicating that altered nuclei are more easily discriminated when irradiated with CI, confirming their higher effectiveness in the targeted region [4]. Moreover, given that the REF class is the same for both binary comparisons, the higher values of *PREC* in the case of CI-REF endorse the same conclusion, i.e., the modified nuclei are more easily discriminated when irradiated with CI.

Depending on CI or XR radiation type, the sub-images derived from HSIs carry optical changes across distinct intervals, i.e., 424–436 nm for CI and 436–445 nm for XR respectively, a consequence of the different effects produced by the two types of radiation.

The best classifiers found in this study can be utilized in future analyses as tools for classifying images from new experiments performed on chondrosarcoma cells. The general procedure proposed here can be applied to any cell line irradiated with any type of radiation to provide quantitative information about the efficiency of irradiation-induced alterations.

Future developments will include cells irradiated at different doses as well as other cell lines to test the capabilities of HSI to detect optical changes in the nuclei of irradiated cells. The procedure meets the needs of the biomedical sector for automated methods capable of capturing and processing large amounts of data to compute irradiation efficiency without biochemical markers and without sophisticated and expensive preparation protocols.

## Data availability statement

Data associated with this article is available at https://doi.org/10.7910/DVN/ATIPXT. More detailed information is available at request to the corresponding author.

## Funding

The in vitro experiments were done at IFIN-HH and CEA for which we acknowledge the Romanian Ministry of Research, Innovation, and Digitalization through: 543PED/2019 and PN 23210202 F1P1. Irradiations with carbon ions were performed at Ganil France using beam time obtained under the experiments nr P1304–H of the iPAC 2021 cal l. Part of this research was supported by 10.13039/501100006595UEFISCDI National Funding Agency (Romania) through the project 576PED/2022. Hyperspectral imaging on CytoViva equipment was possible to be done due to European Regional Development Fund through Competitiveness Operational Program 2014–2020, Priority axis 1, Project No. P_36_611, MySMIS code 107066, Innovative Technologies for Materials Quality Assurance in Health, Energy and Environmental—Center for Innovative Manufacturing Solutions of Smart Biomaterials and Biomedical Surfaces—INOVABIOMED.

## Ethical statement

Our work involves no humans or animals.

Our study includes cell lines.

In the manuscript we noted the name, source, supplier, catalogue number of the cell line used in our study.

The experiments have been performed with mycoplasma-free cells.

Commercial immortalized human cell lines were used in full compliance with ethical and written informed consent requirements.

## CRediT authorship contribution statement

**Raluca D. Negoita:** Writing – original draft, Visualization, Investigation, Formal analysis. **Mihaela A. Ilisanu:** Writing – original draft, Software. **Ionela N. Irimescu:** Writing – original draft, Investigation, Formal analysis. **Roxana C. Popescu:** Writing – original draft, Methodology, Investigation, Formal analysis, Conceptualization. **Mihaela Tudor:** Methodology, Investigation, Formal analysis. **Mona Mihailescu:** Writing – review & editing, Visualization, Validation, Supervision, Data curation, Conceptualization. **Eugen N. Scarlat:** Writing – review & editing, Validation, Supervision, Data curation. **Ana M. Pleava:** Visualization, Investigation. **Anca Dinischiotu:** Writing – review & editing, Supervision, Data curation, Conceptualization. **Diana Savu:** Writing – review & editing, Supervision, Project administration, Methodology, Funding acquisition, Conceptualization.

## Declaration of competing interest

The authors declare that they have no known competing financial interests or personal relationships that could have appeared to influence the work reported in this paper.
